# Chikungunya virus infection induces ultrastructural changes and impaired neuronal differentiation of human neurospheres

**DOI:** 10.3389/fmicb.2023.1152480

**Published:** 2023-05-11

**Authors:** Thaíse Yasmine Vasconcelos de Lima Cavalcanti, Elisa de Almeida Neves Azevedo, Morganna Costa Lima, Karina Lidiane Alcântara Saraiva, Rafael Freitas Oliveira Franca

**Affiliations:** ^1^Department of Virology and Experimental Therapy, Instituto Aggeu Magalhães, Fundação Oswaldo Cruz/Fiocruz, Recife, Brazil; ^2^Core Technology Platforms, Instituto Aggeu Magalhães, Fundação Oswaldo Cruz/Fiocruz, Recife, Brazil

**Keywords:** Chikungunya virus, neuronal differentiation, apoptosis, neurosphere culture, ultrastructural analysis

## Abstract

Chikungunya virus (CHIKV) is an arthropod-borne virus recently associated with large outbreaks in many parts of the world. Infection is typically manifested as a febrile and self-limited illness, characterized by joint pain and myalgia, albeit severe neurological manifestations are also reported. Although CHIKV is not recognized as a truly neurotropic virus, neurons, astrocytes, and oligodendrocytes are susceptible to infection *in vitro*. Here we employed a model of 3D cell culture to obtain neurospheres from ATRA/BNDF differentiated human neuroblastoma cells. We demonstrate that CHIKV is able to establish a productive infection, resulting in ultrastructural changes in cell morphology and impaired neuronal differentiation. Ultrastructural analysis of neurospheres infected with CHIKV during neuronal differentiation revealed diminished neuron dendrite formation, accumulation of viral particles associated with the plasma membrane, numerous cell vacuoles, and swollen mitochondria. Apoptotic cells were significantly increased at 72 h post-infection. Compared to Zika virus, a well-characterized neurotropic arbovirus, CHIKV infection resulted in a more discrete, albeit detectable upregulation of IL-6 levels. Finally, we found that CHIKV infection resulted in an altered profile expression, mainly downregulation, of a group of transcription factors named Hox genes. Altogether our findings highlight important features of CHIKV in the CNS, as well as the feasibility of neurospheres as robust experimental models that can support further studies for novel pharmacological interventions.

## Introduction

Chikungunya fever is an infection caused by the Chikungunya virus (CHIKV), an Alphavirus of the *Togaviridae* family that is transmitted to humans through mosquitoes, mainly *Aedes albopictus* and *Ae. agypti*. Clinically it manifests as acute fever and skin rash, together with disabling arthralgia, arthritis, and fatigue (1). Although CHIKV is not considered a fully neurotropic virus, based on its limited capacity to invade the neural tissue and replicate in the CNS, sporadic cases of neurological manifestations have been reported from different outbreaks. In fact, during the 2005 La Reunion outbreak, CHIKV was associated with neurological diseases in neonates. At least 10 cases of neonates infected with CHIKV with confirmed vertical transmission presenting neurological complications were reported. Of these, 9 (90%) neonates presented neurological manifestations consisting of encephalopathy, a serious condition that can cause temporary or permanent brain damage. The pathological main findings were characterized by brain swelling and cerebral hemorrhage. From these, four children evolved towards persistent disabilities (2). In fact, occasional detections of viral RNA in the CNS of patients presenting neurological diseases reinforced the findings that CHIKV disease is not so rarely associated with neurological infection (3). In mice, CHIKV experimental infection results in virus dissemination to other tissues including the CNS, where it specifically targets the choroid plexuses and the leptomeninges (4). Moreover, the virus is able to infect human astrocytes inducing apoptosis at 48 h post-infection, loss of mitochondrial membrane potential, nuclear condensation, and visible cytopathic effects in a dose and time-dependent manner (5).

One of the first reports to address the potential of CHIKV to infect neuronal cells showed that mouse neurons can be experimentally infected with CHIKV, resulting in extensive cell damage when a high viral dose was employed (6). More recently, human neuroblastoma cells were found to be permissive to CHIKV infection, resulting in the upregulation of active caspase-3, cleavage of PARP, and translocation of cytochrome-c, characteristic features of apoptosis (7). *In vivo*, CHIKV infection induces morphometric changes and innate immune activation in astrocytes. When cynomolgus macaques (*Macaca fascicularis*) were infected with the CHIKV La Reunion strain, a peak viremia between 4.0 and 6.0 log10 PFU/ml was documented at 2 days post-infection. Additionally, white matter astrocyte hypertrophy was noted (8). In young mice, CHIKV infects neurons leading to the upregulation of many cell death-associated genes, inducing an inflammatory response characterized by the upregulation of cytokines and chemokines. Interestingly, cell death pathways induced by CHIKV infection were found to be mainly apoptosis and pyroptosis (a cell death process triggered by proinflammatory signals, being associated with inflammation) (9). Altogether these studies show that CHIKV in fact enters the brain, causing a disease characterized by extensive neuroinflammation.

Neuronal cells can grow either as monolayers of substrate-anchored cells or as suspended aggregates, resulting in the formation of spherical structures called neurospheres. Neurospheres can be cultivated from a variety of normal, genetically altered, cell lineages or pathological tissue explants, allowing the study of neurogenesis, cell differentiation, and even neuropathogenesis (10). Human cell lines are routinely employed at many different research groups, being a powerful model and easy to apply for the study of several aspects of viral infections. SH-SY5Y cells are derived from human neuroblastoma and it is often used as an *in vitro* model of neuronal function and differentiation (11). Furthermore, these cells are susceptible to a wide range of viruses, representing an important tool to neuropathogenesis studies (12, 13). Here, we aimed to characterize CHIKV infection in human neurospheres to model the impact of virus infection during neuronal differentiation. We developed a model of 3D cell culture, employing SH-SY5Y cells grown in the presence of all-trans-retinoic acid (ATRA) and brain-derived neurotrophic factor (BDNF) to achieve fully differentiated neurons, originating neurospheres with mature neuronal structures. Neurospheres were infected with CHIKV during differentiation, showing permissiveness to virus replication. Interestingly, infection resulted in diminished neuron differentiation, apoptosis, and upregulation of proinflammatory markers. These findings provide important insights into CHIKV infection and offer a valuable model for studying the virus’s neurological effects and identifying potential therapeutic targets.

## Materials and methods

### Cell culture and viruses

Human neuroblastoma cell line SH-SY5Y (ATCC^®^ CRL-2266^™^) was grown in Minimum Essential Medium (MEM) supplemented with Ham’s F12 Nutrient Mixture, 10% (*v*/*v*) fetal bovine serum (FBS), 0.1 mM MEM non-essential amino acids, 1 mM sodium pyruvate, 2 mM L-glutamine, 100 U/ml penicillin, and 0.1 g/ml streptomycin, at 37°C with 5% CO_2_ environment. The ZIKV PE243 strain was originally isolated in C6/36 cells from a patient with symptoms of febrile illness as previously reported (14). CHIKV was previously established in our laboratory by isolation from a local case of infection. Viruses were further propagated by conducting four passages in Vero E6 cells. Viral stocks were clarified by centrifugation (2,000 g at 4°C for 10 min), filtered in Millipore Express^®^ PES Membrane Filters (0.22 μm), and stored at −80°C. Virus titer was determined by plaque assay in Vero E6 cells.

### Generation of neurospheres from SH-SY5Y cells

**Figure 1:**
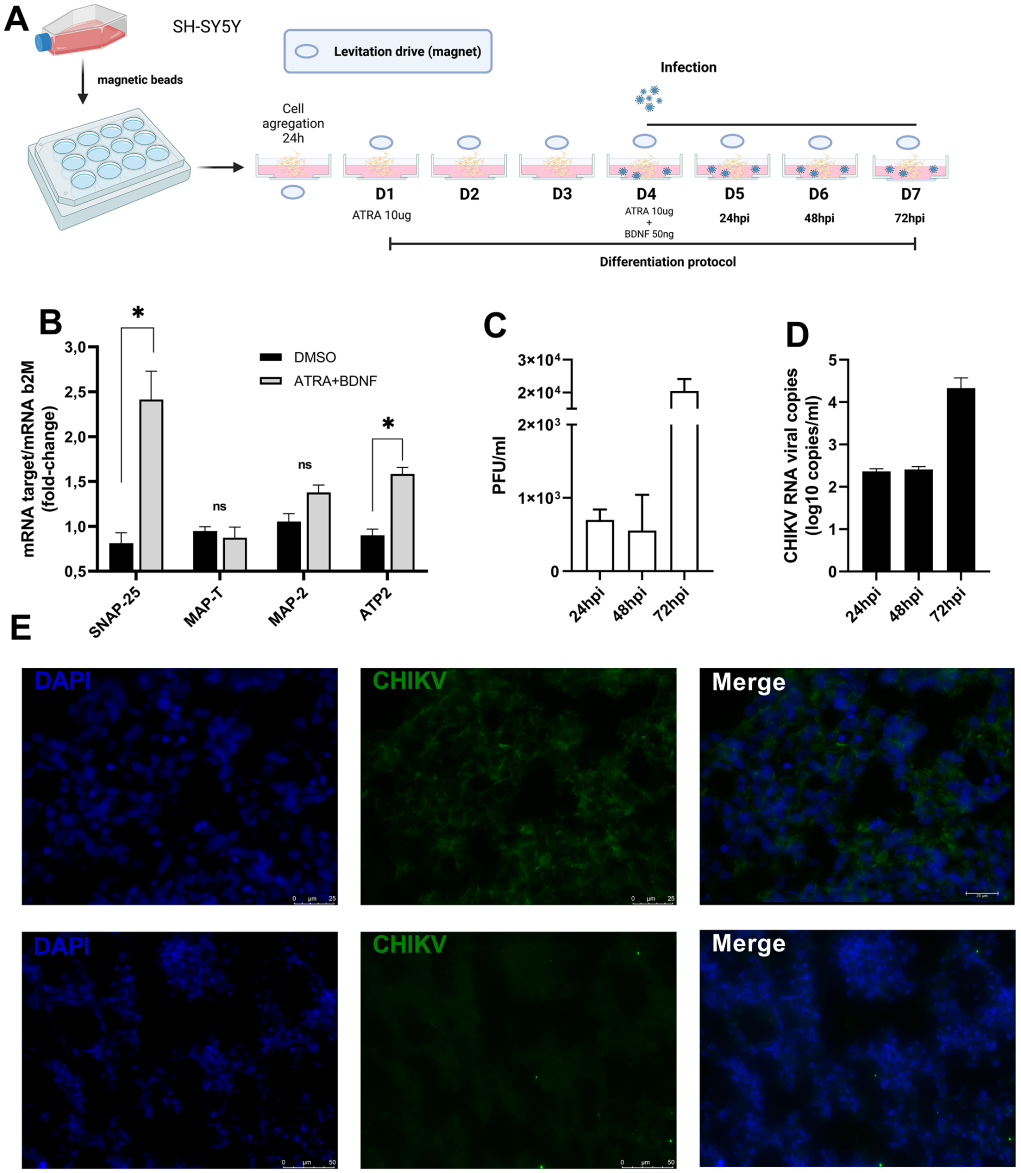
SH-SY5Y neurosphere schematics and characterization. **(A)** Neurospheres 3D culture schematic, SH-SY5Y differentiation, and infection protocol. ATRA—all-trans retinoic acid. BDNF brain-derived neurotrophic factor. Complete differentiation was achieved on day 7. D—day of differentiation **(B)** Relative expression of mRNA for SNAP-25, MAP-T, MAP-2, and ATP2 at 7 days post-differentiation. Fold change relative mRNA expression was calculated by the 2^−ΔΔ^ Ct using the beta-2-microglobulin (b2M) as an endogenous control gene (NS non-significant *p < 0.05 Paired *t*-test). **(C)** Virus titers (PFU/ml) were obtained from infected supernatant neurospheres at 24, 48, and 72 h post-infection, titers were obtained from triplicate experiments and are displayed by error bars (mean ± SD). **(D)** CHIKV viral RNA detection by RT-qPCR in the supernatant of infected neurospheres, time post-infection as described in the figure legends. Data is representative of three independent experiments. **(E)** CHIKV protein detection from mock and CHIKV-infected neurospheres. Cells dissociated from neurospheres were stained with the anti-CHIKV monoclonal antibody (A54Q) at 48 h post-infection. Cell nuclei were counterstained with DAPI in blue. Contrast-phase contrast microscopy imaging. Data indicate the mean and SD of experiments performed in triplicate (*n* = 3). Magnification 100×.

Neurospheres were generated by a 3D cell culture method employing levitating magnetic fields. Briefly, SH-SY5Y cells were grown in regular tissue flasks, and on the next day, the cells were trypsinized and adjusted to 1.5 × 10^5^ cells/ml. Cells were then incubated with 15ul of Nano Shuttle-PL beads (Greiner Bio-One, Kremsmünster, Austria) and seeded in 24 well plates. The plates were then incubated at 37°C in a 5% CO_2_ environment with the magnetic concentration drive (Greiner Bio-One, Kremsmünster, Austria) inserted at the bottom of the plates for 24 h. On the next day, plates were checked and the concentration drive (bottom) was changed by the magnetic levitation drive (top), allowing cells to grow in a levitating magnetic field. On the first day of incubation with the levitation drive, the cells were treated with ATRA (all-trans-retinoic acid) 10uM and incubated at 37°C in a 5% CO_2_ environment, for clarity we defined this as day 1 of neuronal differentiation. On day 4 plates were checked and ATRA MEM/F12 media was replaced with fresh MEM/F12 media containing ATRA 10uM and BDNF (brain-derived neurotrophic factor) 50 ng/ml. Cells were then infected with CHIKV or ZIKV (multiplicity of infection MOI = 1), just after media change on day 4. Plates were then incubated at 37°C in a 5% CO_2_ environment and morphometric analyses were performed by transmission and scanning electron microscopy on day 7, as described below. A schematic for neurosphere generation is provided in Figure 1A.

### Electron microscopy sample preparation

Mock and CHIKV-infected neurospheres were grown as described above and then harvested at 7 days post differentiation with ATRA/BDNF. Neurospheres were fixed in 2.5% glutaraldehyde and 4% formaldehyde in 0.1 M sodium cacodylate buffer, pH 7.4. For scanning electron microscopy (SEM) the specimens were washed in PBS and post-fixed for 30 min in 1% osmium tetroxide (OsO₄). After that, they were dehydrated in ethanol series and critical point-dried with liquid CO_2_. The specimens were coated with a thin layer of gold and then observed with a Jeol JSM-5600 electron microscope, operated at 15 kV. For transmission electron microscopy (TEM) the fixed neurospheres were washed in 0.1 M cacodylate buffer, pH 7.4, and post-fixed in a solution containing 1% OsO4, 2 mM calcium chloride, and 0.8% potassium ferricyanide in 0.1 M cacodylate buffer. The samples were counterstained in 2.5% uranyl acetate, dehydrated in acetone series, embedded in EMbed 812 resin. 70 nm sections were collected on nickel grids, and stained with 5% uranyl acetate and 1% lead citrate. The cells were visualized with an FEI Tecnai G2 Spirit BioTwin transmission electron microscope, operated at 120 kV.

### CHIKV replication kinetics by real-time PCR

Supernatants from mock and CHIKV-infected neurospheres were harvested at the indicated time points after infection and the viral RNA was extracted using the QIAamp Viral RNA Mini kit (QIAGEN, Hilden, Germany). Quantitative RT-PCR (RT-qPCR) reactions were performed with the GoTaq^®^ Probe 1-Step RT-qPCR (Promega, Madison WI, United States), and virus-specific primers and probes were employed, as previously described (15). Reactions were performed with the QuantStudio 5 Real-Time PCR System (Thermo Fisher Scientific, Waltham, MA, United States) under the following cycling conditions: 45°C for 10 min; 95°C for 5 min; 95°C for 5 s; and 60°C for 45 s in a total 40 cycles.

### Annexin V staining and flow cytometry

Mock and CHIKV-infected neurospheres were grown as described and then harvested at 48 h post-infection. Neurospheres were washed with PBS, dissociated with Accutase^™^ Cell Detachment Solution (BD Biosciences, Franklin Lakes, NJ, United States), and stained with Annexin V (FITC) and 7-Aminoactinomycin D (7-AAD), according to the manufacturer’s instructions. All FCM analyses were performed using BD FACSAriaIII Cell Sorter (BD Biosciences, Franklin Lakes, NJ, United States) and data analysis was performed using the FlowJo (TreeStar, Woodburn, OR, United States) software.

### CHIKV protein labeling

Mock and CHIKV-infected neurospheres were harvested at the indicated time points. Subsequently, they were fixed, blocked, and frozen for histological sections. The samples were then treated following the same specifications used for phalloidin labeling. Intracellular antibody labeling was performed employing a CHIKV-specific monoclonal antibody (A54Q) (Thermo Fisher Scientific) and a FITC-conjugated goat anti-mouse IgG secondary antibody (Sigma-Aldrich). After incubation, cells were washed extensively with PBS, and slides were mounted with Fluoromount-G^™^ mounting medium with DAPI (2-(4-Amidinophenyl)-6-indolecarbamidine), images were captured on a Leica immunofluorescence microscopy DMi8.

### Cytokines

Soluble cytokines and chemokines were quantified in the supernatant of infected and control neurospheres cultures by the kit Cytometric Bead Array (CBA) Human Inflammatory Cytokine and Human Chemokine Kit (BD Biosciences, San Diego, CA, United States), following the manufacturer’s instructions.

### Gene expression—TaqMan array

Mock, CHIKV and ZIKV-infected neurospheres were harvested at the indicated time points after infection (as stated in figure legends) and processed for total RNA extraction using the RNeasy Micro Kit (QIAGEN, Hilden, Germany). cDNA synthesis was performed using the High-Capacity cDNA Reverse Transcription Kit (Thermo Fisher Scientific, Waltham, MA, United States), according to the manufacturer’s instructions. The quality of cDNA samples was evaluated using the NanoDrop^™^ spectrophotometer (Thermo Fisher Scientific, Waltham, MA, United States). cDNA samples were quantified using the QubitTM dsDNA HS Assay Kit (Thermo Fisher Scientific, Waltham, MA, United States). Expression analysis was performed using TaqMan^®^ *Array Human hox Genes 96-well Plate* (Thermo Fisher Scientific, Waltham, MA, United States). We loaded cDNA at a concentration of 3.5 ng per reaction. All qPCR reactions were performed using the QuantStudio 5 Real-Time PCR System (Thermo Fisher Scientific, Waltham, MA, United States) Gene expression analysis was performed using the 2[−∆∆CT] method, and values for each condition were considered as gene expression fold-change. Heatmaps were constructed using GraphPad Prism (Graph Pad Software Inc., San Diego, CA, United States) software.

### Statistical analysis

Data are shown as mean ± standard deviation (SD). Statistical analysis was performed using Paired *t*-test as indicated in the figure legends. All statistical analyses were performed using GraphPad Prism 9 software (GraphPad Software, Inc., La Jolla, CA, United States), and the results were considered significant when the *p*-value was below 0.05.

## Results

### CHIKV infects human neuron-differentiated neurospheres

Our protocol was based on a 3D cell culture model. On the first day, undifferentiated SH-SY5Y cells were dissociated from cell flasks and then incubated with nanoshuttle magnetic beads. Cell-complexed beads were seeded at a concentration of 1.10^5^ cells/ml in 24 wells plates and incubated for up to 24 h at 37°C in a 5% CO_2_ environment under an inferior (bottom) magnetic field to induce cell aggregation. After 24 h the magnetic field was changed to the superior (top) position of the plates and cell aggregates were treated with ATRA 10 uM to induce neuronal differentiation (this was considered neuronal differentiation day 1). On day 4 cells were again treated with ATRA 10 uM plus BDNF 50 ng/ml and let to differentiate for up to 7 days (Figure 1A). On day 7 after neuronal differentiation with ATRA/BDNF, we observed the formation of more rigid, spherical structures, and characteristic features of neurospheres, when compared to untreated cells (Supplementary Figure S1). Phalloidin-stained neurospheres displayed highly-organized structures, as evidenced by a well-defined actin filaments distribution (Supplementary Figure S2). Next, we addressed neuronal differentiation, as indicated by the expression of neuronal transcripts SNAP-25, MAP-T, MMAP-2, and ATP2. Albeit, only SNAP-25 and ATP2 transcripts were significantly upregulated at 7 days post-differentiation (Figure 1B), we concluded that our protocol resulted in neuron-differentiated cells.

Next, to explore the feasibility of our model, we evaluated the permissiveness to CHIKV infection of our neurospheres model. For this, neurospheres were differentiated as described and infected with CHIKV (MOI = 1) on differentiation day 4 as indicated in Figure 1A. To confirm infection cells were dissociated from neurospheres and stained with a CHIKV-specific monoclonal antibody (CHIKV A54Q). No staining was observed from mock-infected cells and at 48 h post-infection (hpi) we detected high numbers of positively stained cells (Figure 1E). Production of increasing titers of infectious virus particles was also confirmed in the infected neurospheres by plaque forming unit assay (PFU), in which higher viral titers were achieved at 72 hpi (Figure 1C). We also detected increasing amounts of viral RNA from neurosphere supernatants from 24 h post-infection onwards, reaching a peak at 72 hpi (Figure 1D). Based on this, we conclude that neuron-differentiated 3D cell cultures (neurospheres) were permissive to CHIKV experimental infection and supported viral replication.

### CHIKV infection leads to impaired neurite formation

**Figure 2:**
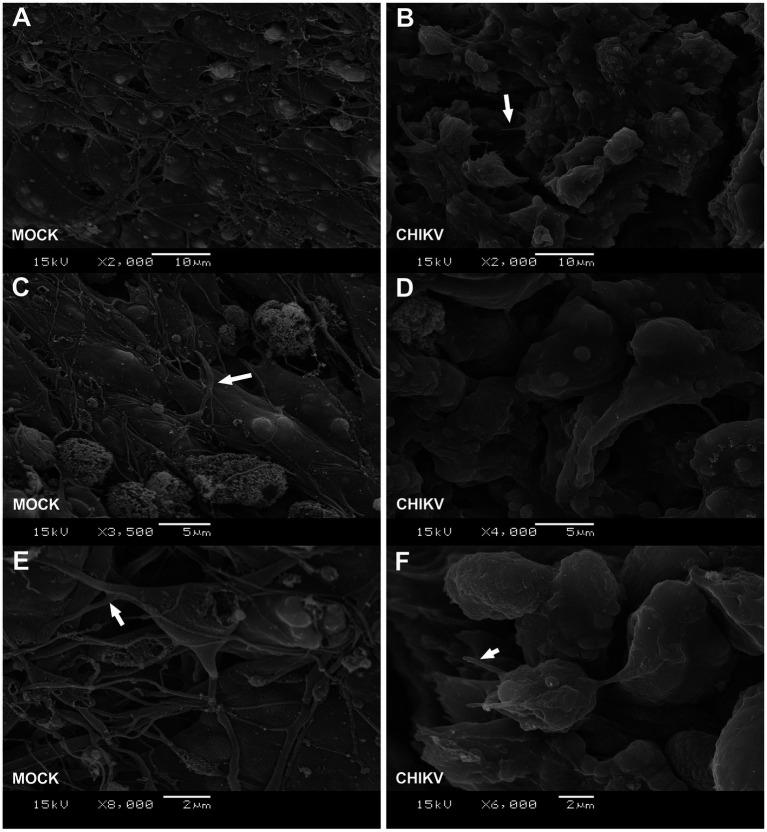
Neurospheres SEM ultrastructure. **(A,C,E)** Scanning electron microscopy (SEM) of mock-infected neurospheres. White triangular arrows indicate neurites extended out from cell bodies. **(B,D,F)** Scanning electron microscopy (SEM) images of CHIKV (MOI = 1) infected neurospheres at 48 h post-infection. White triangular arrows indicate damaged neurites. Neurospheres were cultured for 7 days and infected at differentiation day 4, as described in Figure 1A. SEM image magnification and scale bars are shown in the figure legends (bottom).

Given that we determined the permissiveness of neurospheres to CHIKV, we next aimed to explore the alterations in cell morphology resulting from infection. To achieve this, neurospheres were infected with CHIKV (MOI = 1) at differentiation day 4 and allowed to fully differentiate into neurons until day 7. Neurospheres were then analyzed by scanning electron microscopy (SEM) at 72 hpi, where we observed massive ultrastructural changes in CHIKV-infected neurospheres. SEM imaging of the neurosphere surfaces showed an abundance of round-shaped cells, along with the presence of damaged neurites and reduced neurite formation (neurite length) compared to mock-infected neurospheres (Figures 2A–F). Albeit we observed dimindshed neurit formation the size of CHIKV-infected neurospheres was unaltered, when compared to mock-infected neurospheres, at 3 days post infection (Supplementary Figure S3). These findings suggest that CHIKV infection impairs neuronal differentiation.

### Ultrastructural changes in neurospheres induced by CHIKV infection

**Figure 3:**
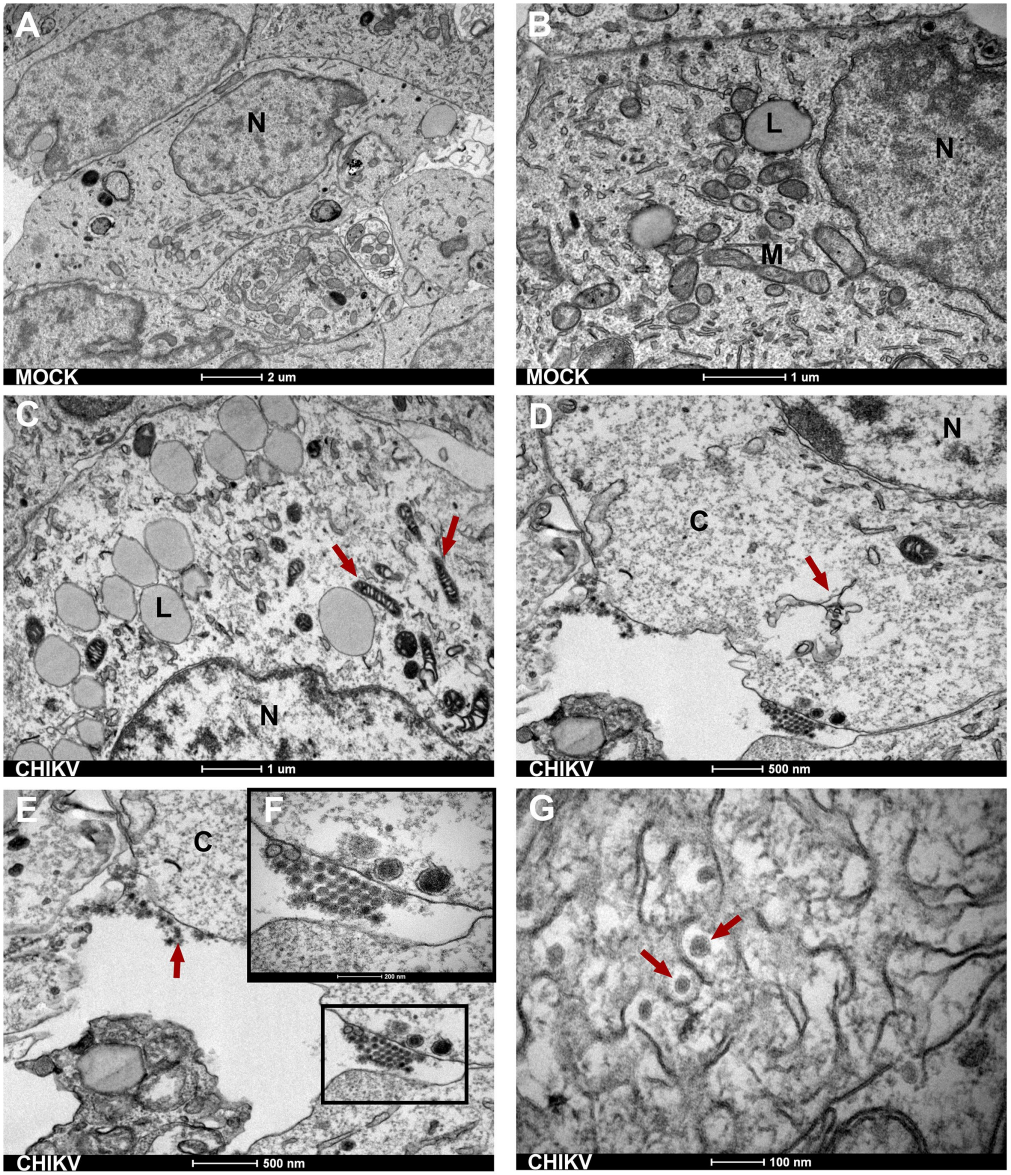
Neurospheres TEM ultrastructure. **(A,B)** Electron micrographs of mock-infected cells show normal morphology of SH-SY5Y cells with a normal nucleus (N) showing normal morphology of cytoplasmic organelles-mitochondria (M) and rough endoplasmic reticulum (with triangular arrow). Bars 1 μm and 2 μm. **(C)** Red arrows indicate electron-dense mitochondrial. Bar 1 μm. **(D)** Infected neurospheres with damage to the endoplasmic reticulum (red triangular arrow) Bar 500 nm. **(E,F)** CHIKV-infected neurospheres show disrupted cell membranes and the presence of viral aggregates at the cell membrane (black square). Bar 500 nm. **(G)** Intracytoplasmic membranous viral aggregates showing spherical viral particles with an electron-dense inner core (nucleocapsid) and outer envelope. Bar 100 nm. N, nucleus; L, lipid droplet; M, mitochondria; C, cytoplasm.

Next, to better explore the morphological changes induced by CHIKV infection we analyzed the neurospheres by transmission electron microscopy (TEM). TEM evidenced the presence of broad cellular ultrastructural changes observed in the infected neurospheres. Initially, for comparison, mock-infected neurospheres showed preserved organelles and membranes, and an unaltered cellular architecture. More precisely, no cytoplasmic vacuolation, mitochondria, and rough endoplasmic reticulum alteration or nuclear degradation were observed in mock-infected neurospheres (Figures 3A,B). When neurospheres were infected with CHIKV we observed the presence of swollen and electron-dense mitochondrial structures (Figure 3C red arrows). In Figure 3D, it can be observed that the infected neurospheres display damaged endoplasmic reticulum structures (indicated by the red arrows) and an electron-lucent cytoplasm. The electron-lucent appearance of the cytoplasm suggests that viral replication may have caused the depletion of organelles, leading to a loss of cellular integrity. We also detected an accumulation of viral particles (viral aggregates) adjacent to cell membranes (Figures 3E,F). At 72 hpi, we observed the accumulation of intracellular viral particles with electron-dense nuclei surrounded by a viral envelope formed by the endoplasmic membrane. It is also possible to observe scattered intracytoplasmic viral particles (Figure 3G), a common feature of the CHIKV replication cycle as previously reported by independent groups (16, 17).

### CHIKV infection leads to apoptosis of neurospheres

**Figure 4:**
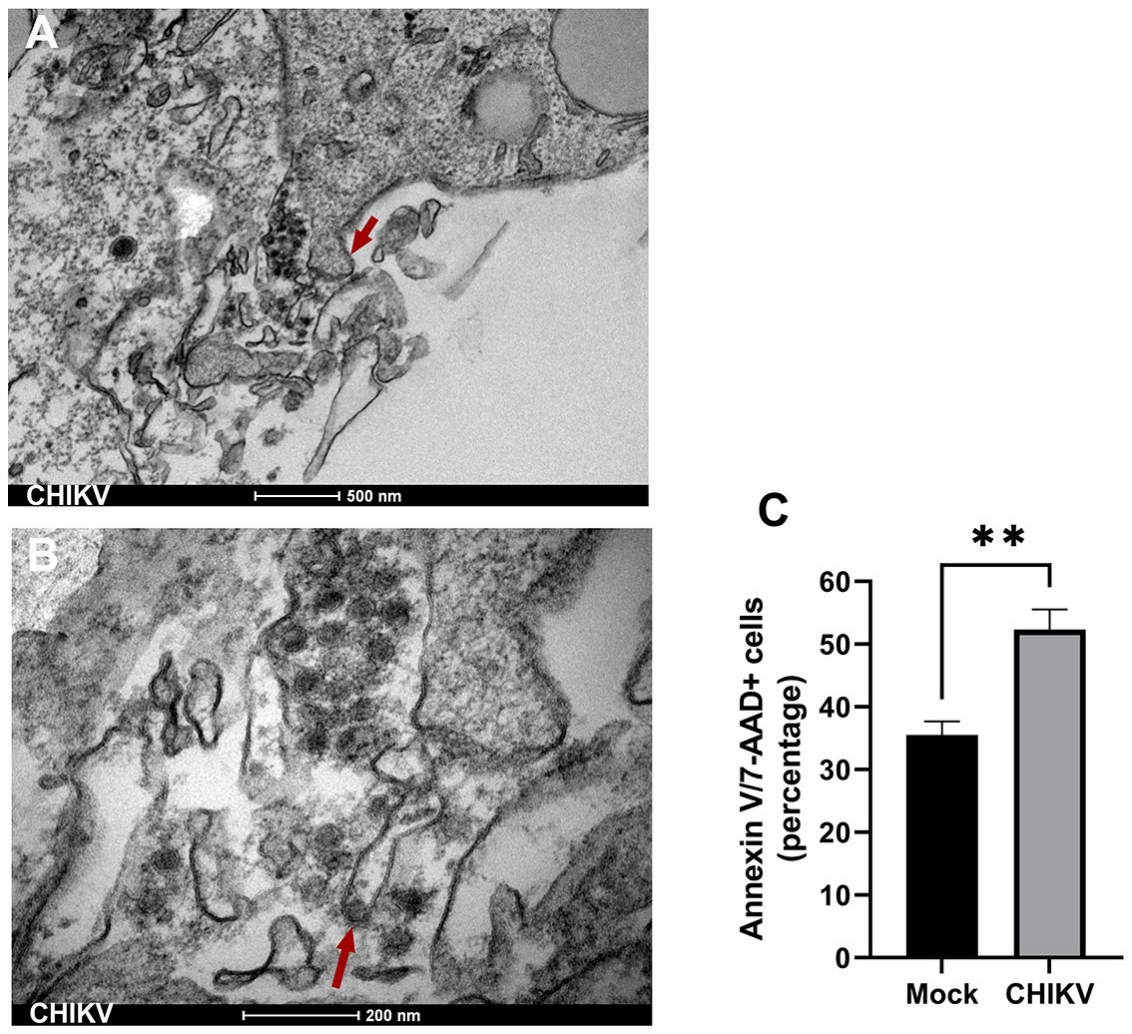
Apoptosis induced by CHIKV infection. **(A)** Transmission electron microscopy (TEM) of CHIKV-infected neurospheres showing the presence of membrane blebs (triangular red arrow), released membrane vesicles (apoptotic bodies), and accumulation of viral particles adjacent to membrane protrusions. Bar 500 nm. **(B)** TEM higher magnification shows a viral particle budding from a cell membrane (triangular red arrow). Bar 200 nm. **(C)** Frequency of Annexin V/7-AAD positive stained cells from mock and CHIKV infected neurospheres. Data indicate the mean and SD of experiments performed in triplicate (*n* = 3). Groups were compared by Paired *t*-test (**p < 0.01) using the software GraphPad Prism 9.

Since altered mitochondrial structures are an indicator of cellular stress and cell death, we performed a more detailed observation of infected neurospheres. TEM of CHIKV-infected neurospheres showed the presence of nuclear condensation (not shown), along with many membrane blebs and membrane-bound apoptotic bodies, which are classical features of apoptosis (Figure 4A). Interestingly, these membrane protrusions were associated with the presence of abundant viral particles. More precisely, we observed viral aggregates being released from infected cells by cell membrane protrusions, and we could also observe protrusions carrying single particles at the apical tip (Figure 4B). Altogether, these data suggest that CHIKV damages cells directly during the budding process required to acquire the virus envelope from the cell membrane. Thus, to evaluate whether CHIKV infection results in apoptosis, we stained neurospheres with Annexin V/7-AAD at late infection time points (72 hpi). We detected significantly increased levels of Annexin V/7-AAD positive cells from CHIKV-infected neurospheres at 72 hpi, confirming extensive cell death induced by CHIKV infection (Figure 4C).

### Inflammatory and transcriptional profile of CHIKV-infected neurospheres

**Figure 5:**
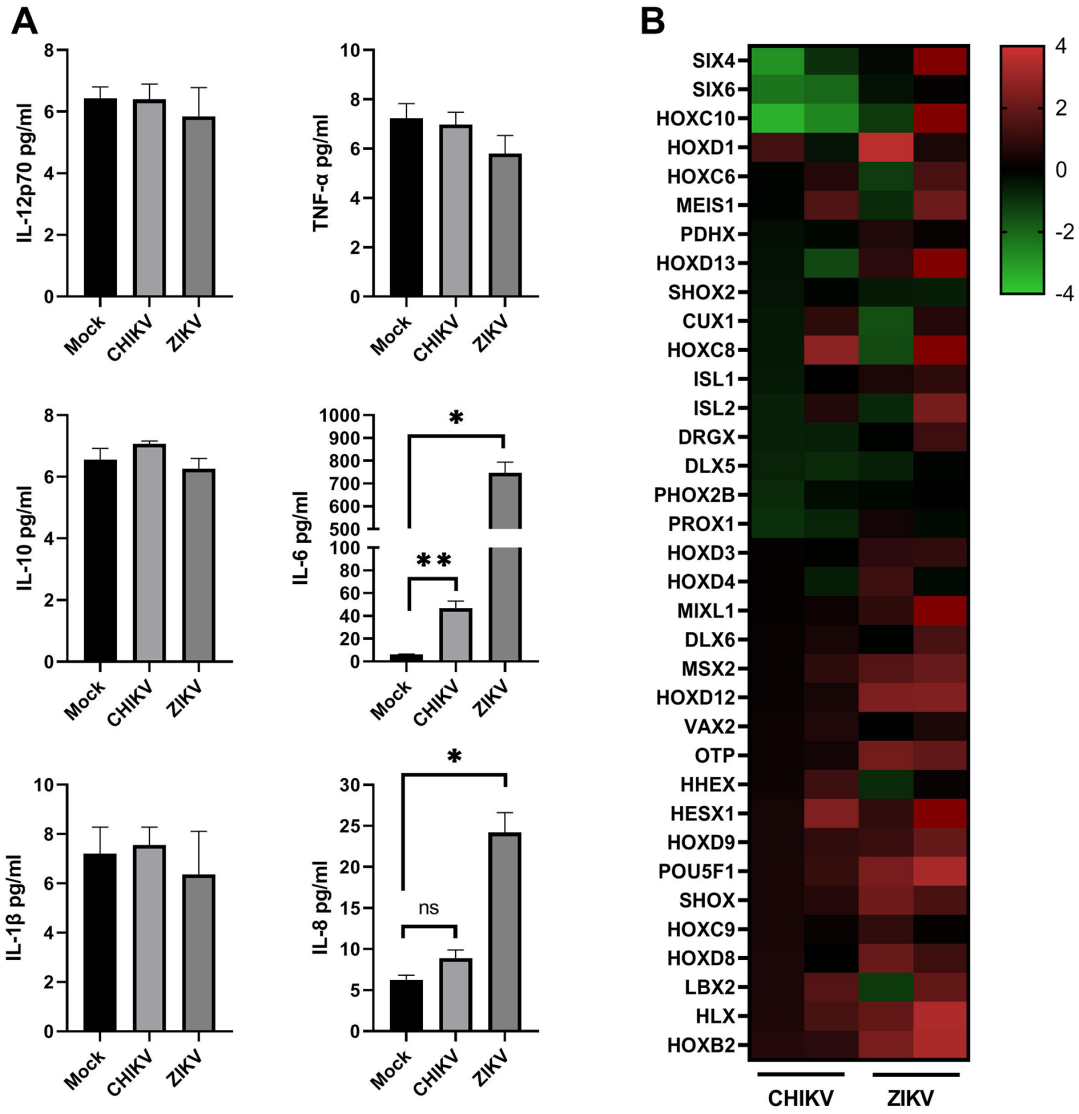
Inflammatory and transcriptional profile of infected neurospheres. **(A)** Levels of proinflammatory cytokines from mock, CHIKV (MOI = 1), and ZIKV-infected (MOI = 1) neurospheres supernatant. Individual cytokine levels (as indicated in figures) were measured in neurospheres cell-free supernatants at 72 h post-infection. Data indicate the mean and SD of experiments performed in triplicate (*n* = 3), values are represented in picograms per milliliter (pg/mL). Groups were compared by Paired t-test (*p < 0.05, **p < 0.01) using the software GraphPad Prism 9. **(B)** Transcriptional changes induced by CHIKV and ZIKV infection of neurospheres. Values are expressed as Log_2_ fold change relative to mock-infected neurospheres. Experiments were performed in duplicate (each column represents a single experiment).

Given that neurospheres were susceptible to CHIKV infection, with a significant loss in cell viability and massive ultrastructural changes, we aimed to analyze the transcriptional changes and the inflammatory profile induced by the virus. For this, we infected the neurospheres during the neuronal differentiation process with CHIKV and compared the inflammatory and transcriptional profile induced by the infection with ZIKV, a well-characterized neurotropic arbovirus. At 72 hpi we detected significantly increased levels of the pro-inflammatory cytokine IL-6 in the supernatant of CHIKV-infected neurospheres, albeit to a lesser extent when compared to ZIKV-infected neurospheres (Figure 5A). IL-8 levels from CHIKV-infected neurospheres were only slightly increased. Altogether these data show that CHIKV infection leads to immune activation of mature neurons.

Finally, to evaluate if the impaired neuronal differentiation would be associated with an altered transcriptional profile of CHIKV-infected neurospheres we analyzed the global expression of Hox genes, a set of transcription factors that are highly expressed in the CNS, where they have critical functions such as neuronal development and circuit organization (18). Overall, CHIKV infection resulted in pronounced down-regulation of several Hox genes, whereas ZIKV infection preferentially leads to up-regulation of these transcription factors. Among differentially expressed genes, we found that sine oculis homeobox (SIX) family members SIX4, and SIX6 were downregulated. Members of the SIX family transcription factors control gene expression to promote the self-renewal of progenitor cell populations and govern mechanisms of cell differentiation (19). We also detected that homeobox protein Hox-C10 (HOXC10) was strongly downregulated at 48 hpi (Figure 5B). We observed many other downregulated Hox genes from CHIKV-infected neurospheres, albeit to a lesser extent, confirming that CHIKV infection alters neuronal differentiation in human neurosphere cultures.

## Discussion

Since the La Réunion outbreak in 2005, severe presentations of CHIKV infection with neurological involvement and fulminant cases of hepatitis have been reported (20). The most common neurological complications include encephalitis, myelopathy, peripheral neuropathy, myeloneuropathy, and myopathy. Furthermore, the presence of virus-specific IgM antibodies, with some sporadic cases of virus detection in CNS reinforces the neurological potential of CHIKV (21). Although the virus possesses the potential to induce neurological complications, as documented among different outbreaks, its neuropathogenesis mechanisms are poorly explored. Here we found that human neurospheres are permissive to CHIKV infection, representing an important model for studying virus-induced pathogenesis in the CNS. Since our experimental approach employed a protocol based on neurosphere infection during the neuronal differentiation process we were able to track virus-induced changes during a critical stage of cell development. Our results suggest that CHIKV infection results in extensive cell death and diminished neuronal differentiation, evidencing the deleterious effects of CHIKV in the CNS.

Recently, CHIKV infection was characterized by the human microglial cell line CHME-3, showing that microglial cells are permissive to CHIKV infection, supporting virus replication with the highest viral titers 36 hpi. Interestingly, cell viability was not significantly affected at late time points after infection (22). These findings contrast with our observations, where CHIKV infection of neurospheres derived from human neuroblastoma cells results in extensive morphological changes and cell death. Interestingly, our data is in line with a previous study showing that SH-SY5Y cells are highly infected, resulting in extensive apoptosis characterized by caspase-3 activation, cleavage of PARP, and translocation of cytochrome-c (7). Precisely, we demonstrated that CHIKV-exposed neurospheres displayed extensive ultrastructural changes, characterized by reduced formation of neurites, presence of damaged neurites, and extensive cell apoptosis. However, it remains elusive if the impaired neuronal differentiation observed in our model is a direct effect of virus infection (i.e., interference with the cellular machinery) or is just a consequence of neuroinflammation and cellular apoptosis.

To the present, most of the studies of CHIKV neuropathogenesis focussed on the use of standard-grown monolayer attached neuronal cell lines, these models were mostly based on CHIKV infection of immature neurons (23). In the current study, we explored a more robust model of neuronal pathogenesis, employing human neurospheres to simulate virus infection in the CNS. Although we were not able to define whether the virus preferably infects fully mature neurons, or if it is restricted to cells in the process of differentiation, Dhanwani et al. (7) reported that ATRA differentiated human SH-SY5Y cells were less susceptible to CHIKV-induced cytopathic effects, showing enhanced cell viability when compared to undifferentiated cells. However, it remains elusive whether the CHIKV-induced neuronal damage would be more related to the host inflammatory response than to a direct effect of virus replication on susceptible cells.

As previously reported CHIKV neurological complications are the most common forms of severe disease presentation, accounting for approximately 25% of atypical and 60% of severe cases (24, 25). In addition to this, CHIKV neurological manifestations are much more common in newborns and older individuals (aged >60 years or above), being recently associated with long-term cognitive decline and dementia in elderly people (26). Also, a series of independent studies reported a range of neurocognitive outcomes (albeit contradictory) in perinatally infected children (27–29), however, more studies are necessary to correctly address the causality of CHIKV infection and neurodevelopmental defects. Through this study we brought more data to the topic of the neurological effects of CHIKV infection, showing that neuronal differentiation was impaired in CHIKV-infected neurospheres, this was followed by an inflammatory response characterized by the production of IL-6 and IL-8, which may further aggravate neuronal damage. As extensively explored, IL-6 and IL-8 levels are commonly increased in many CNS infections and/or CNS injuries, and although their effects may have a dual role (also contributing to neurogenesis) IL-6 exposure during neuronal development can result in cell damage and death in developing neurons (30, 31). Whereas IL-8 is frequently associated with neuroinflammation (32, 33) and CNS injury (34).

Recent findings suggest that in addition to their well-known roles at the early embryonic stages, the Hox genes are crucial to later aspects of neuronal circuit development, including stereotypic neuronal migration, axon pathfinding, and topographic mapping of connectivity (35), making it a desirable target to better explore the neurological perturbations induced by CHIKV infection. Here, we compared the profile of Hox genes expression resulting from CHIKV infection to ZIKV infected-neurospheres. Interestingly, we found that CHIKV infection resulted in a more pronounced downregulation of SIX4, SIX6, and HOXC10 genes. This disparity of Hox genes expression among CHIKV and ZIKV could be explained by the rapid accumulation of apoptotic cells induced by CHIKV, starting from 48hpi (data not shown), reaching a peak of 50% apoptotic cells after 72 hpi. Thus, since ZIKV is well-known for its ability to induce cell cycle arrest and delayed neuronal apoptosis (36), contrasting with CHIKV infection that leads to a rapid accumulation of apoptotic cells, we may assume that this marked downregulation of Hox genes may result from rapid and excessive cell death, rather than a direct modulatory effect of CHIKV infection. In fact, like other alphaviruses, CHIKV is highly cytopathic in human cell cultures, and infected cells rapidly undergo apoptotic cell death (37).

Neurospheres are a highly valuable model for studying functional neuronal characteristics and have been employed by several research groups to investigate the biological properties of the CNS. Our model, which involves infecting cells during the neuronal maturation process, allowed us to explore the potential neuropathogenesis of CHIKV during neuronal growth, making our findings particularly relevant in the context of CHIKV infections. However, it is important to note that this study is mainly exploratory and does not specifically identify any molecular processes related to apoptosis or virus replication, such as host factors that regulate infection and apoptosis. Additionally, unlike ZIKV, CHIKV is not typically associated with birth developmental defects, although a number of neurological disorders have been linked to CHIKV infections. Despite these limitations, our findings are significant, as they demonstrate that neurospheres can serve as a useful model for defining the neuropathogenesis of CHIKV.

## Data availability statement

The original contributions presented in the study are included in the article/Supplementary material, further inquiries can be directed to the corresponding author.

## Author contributions

TL conceived the project, performed the experiments, data analysis, and interpretation. RF conceived and supervised the study and performed data analysis and interpretation. TL and RF prepared the manuscript. All authors contributed to the article and approved the submitted version.

## Funding

TL was supported by Vice-Presidência de Educação, Informação e Comunicação (VPEIC) da Fiocruz. This study was financed in part by the Coordenação de Aperfeiçoamento de Pessoal de Nível Superior (CAPES) - Brazil - Finance Code 001. This research was supported by the Fundação de Amparo à Ciência e Tecnologia de Pernambuco/FACEPE (grant number APQ-0055.2.11/16 and APQ-0044.2.11/16), and Conselho Nacional de Desenvolvimento Científico e Tecnológico/CNPq (grant number 439975/2016-6) under RFOF coordination and responsibility.

## Conflict of interest

The authors declare that the research was conducted in the absence of any commercial or financial relationships that could be construed as a potential conflict of interest.

## Publisher’s note

All claims expressed in this article are solely those of the authors and do not necessarily represent those of their affiliated organizations, or those of the publisher, the editors and the reviewers. Any product that may be evaluated in this article, or claim that may be made by its manufacturer, is not guaranteed or endorsed by the publisher.

## Supplementary material

The Supplementary material for this article can be found online at: https://www.frontiersin.org/articles/10.3389/fmicb.2023.1152480/full#supplementary-material
