# Supplementary material for: Chikungunya virus infection induces ultrastructural changes and impaired neuronal differentiation of human neurospheres
Source: Front Microbiol. 2023 May 11;14:1152480. doi: 10.3389/fmicb.2023.1152480 (PMC10213924; doi:10.3389/fmicb.2023.1152480)
Supplement: Supplementary file 1 [file Data_Sheet_1.PDF]

Chikungunya virus infection induces ultrastructural changes and impaired neuronal differentiation of human neurospheres

Thaíse Yasmine Vasconcelos de Lima Cavalcanti[1], Elisa de Almeida Neves Azevedo[1], Morganna Costa Lima [1], Karina Lidiane Alcântara Saraiva[2] and Rafael Freitas Oliveira Franca[1*]

Supplementary Methods

Phalloidin Labeling Differentiated neurospheres were fixed with PBS paraformaldehyde (PFA) 4% and blockaded in Tissue Tek OCT (Optimum cutting temperature) (Compound, Fisher Healthcare) and immediately frozen in liquid nitrogen by dip. The blocks were used for histological sections by the Leica CM1860 cryostat at a temperature of 27°C with the thickness of 10 µm. The samples were permeabilized with PBS (phosphate buffered saline) plus Triton X-100 0.5 % and were washed with PBS. Subsequently the samples were treated with a blocking solution 5% bovine serum albumin (BSA)-PBS. For the labeling of the cellular structure Alexa Fluor 488 phallodin (A12379) (Thermo Fisher Scientific) and the slides were assembled with Fluoromount-G™ mounting medium with DAPI (2-(4-Amidinophenyl)-6-indolecarbamidine), images were captured in a Leica DMi8 immunofluorescence microscopy.

Supplementary Results

On day 7 after neuronal differentiation with ATRA/BDNF, we observed the formation of more rigid, spherical structures, and characteristic features of neurospheres, when compared to untreated cells (Supplementary Figure 1). Phalloidin-stained neurospheres displayed highly-organized structures, as evidenced by a well-defined actin filaments distribution (Supplementary Figure 2).

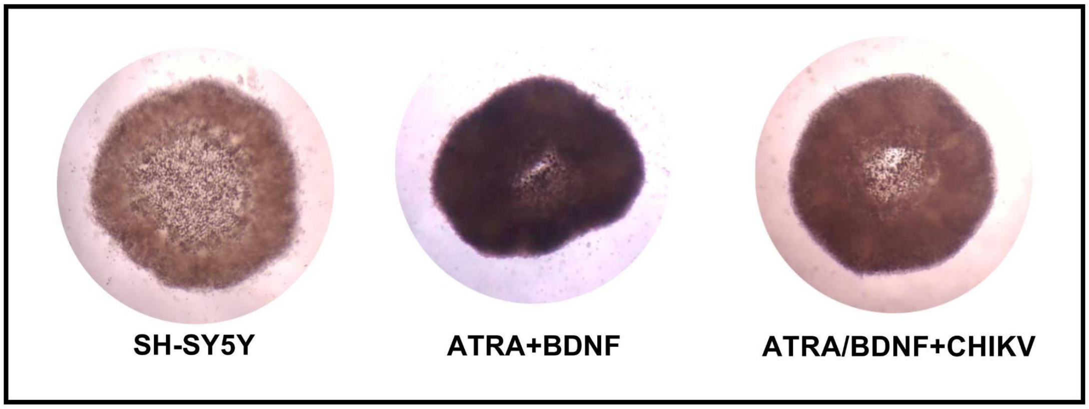

Supplementary Figure 1. Brightfield photomicrographs of Mock and CHIKV-infected neurospheres.

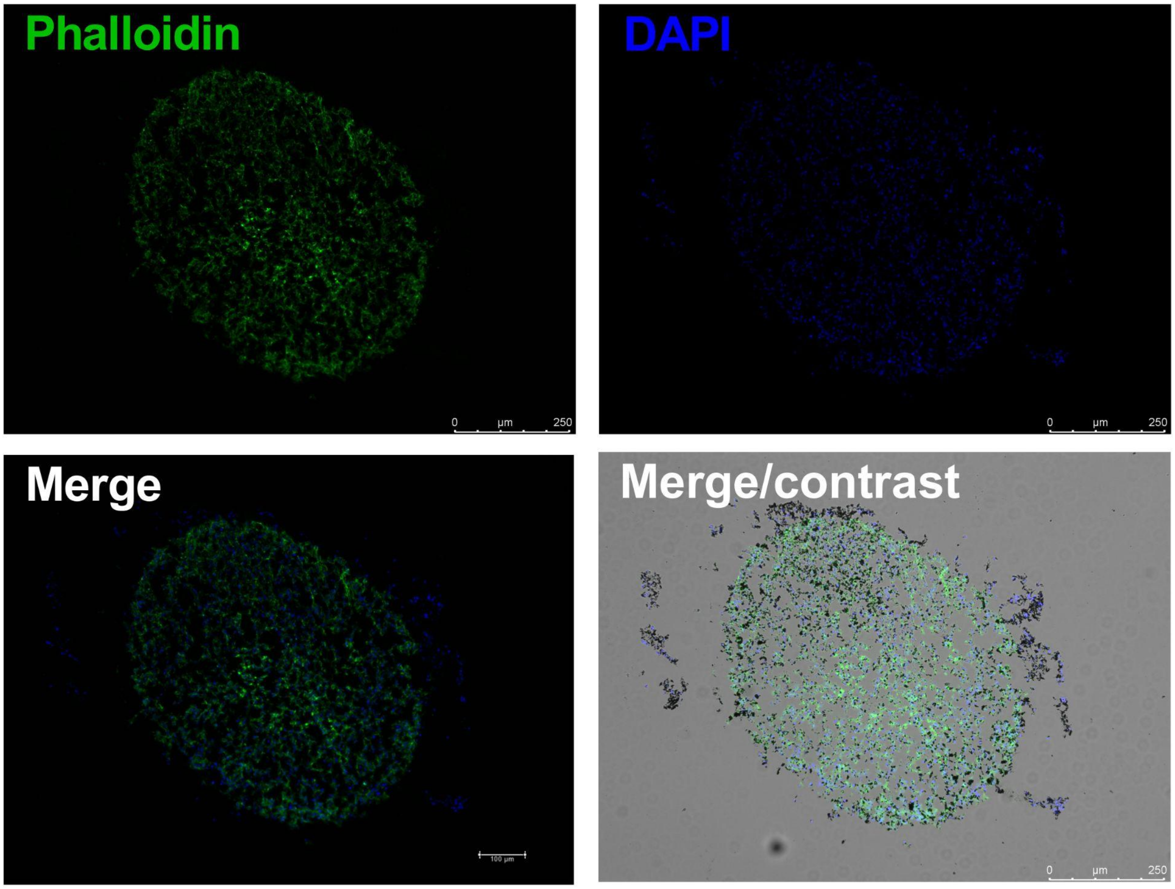

Supplementary Figure 2. Phalloidin staining (green) of actin filaments from fully differentiated neurospheres at 7 days-post differentiation. Cell nuclei were counterstained with DAPI in blue. Magnification 40x.

At three days after infection, both mock and CHIKV-infected neurospheres presented similar area (neurospheres diameter measured in milimiters).

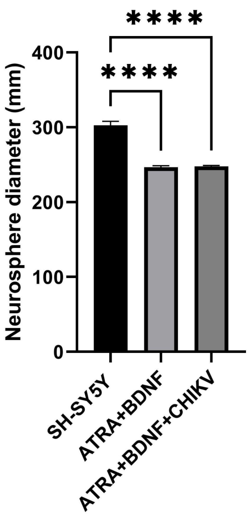

Supplementary Figure 3. Neurosphere diameter (in milimiters) at three days post-infection.
